# Participatory Approaches in Family Health Promotion as an Opportunity for Health Behavior Change—A Rapid Review

**DOI:** 10.3390/ijerph19148680

**Published:** 2022-07-16

**Authors:** Maja Kuchler, Marie Rauscher, Pia Rangnow, Eike Quilling

**Affiliations:** 1 Department of Applied Health Sciences, Hochschule für Gesundheit, University of Applied Sciences, 44801 Bochum, Germany; marie.rauscher@hs-gesundheit.de (M.R.); eike.quilling@hs-gesundheit.de (E.Q.); 2 Department of Health Sciences, Fulda University of Applied Sciences, 36037 Fulda, Germany; pia.rangnow@pg.hs-fulda.de

**Keywords:** family, participatory research, health promotion and prevention of chronic diseases, experience, methods

## Abstract

Background: With their influence on health behavior of children, families are important addressees in health promotion and prevention of chronic diseases. However, they are often difficult to reach, partly due to the open approach of health promotion services. Therefore, they should be addressed directly and be involved in shaping their living environment. The aim is to examine which approaches are used in participatory family health promotion and what practical experiences are made. Methods: A systematic literature search in PubMed, Web of Science, LIVIVO and a supplementary hand search were conducted. Ten of 718 screened publications were analyzed qualitatively. Results: Most included publications applied the community-led participatory approach CBPR. In seven publications, family actors could make decisions at any or all project phases. One finding is that positive effects on desired behavior change and improved health of target groups were observed. Frequently described success factors are the type of interaction, and a common goal. Conclusion: The forms of family participation in health promotion vary widely, with the lack of participatory practices being a major challenge. Family participation is a useful approach in shaping health promotion and should be further developed. This overview provides support for planning future participatory projects with families.

## 1. Introduction

Families play a crucial role in health promotion and prevention of chronic diseases [1]. Families are long-term communities in which individual health behaviors are developed and consolidated [2]. This influence of the family on the health behavior of individual family members runs through all phases of life [3]. Chronic diseases, such as cardiovascular diseases or diabetes mellitus, are among the most common and economically significant health problems in industrialized countries [4]. Risk factors that influence the development of chronic diseases are already evident in childhood and adolescence. Health promotion and primarily prevention is a sensible starting point for combating chronic illnesses because they can prevent, reduce, or delay damage to health [5]. Therefore, the role of the family is particularly evident in the early stages of life, such as pregnancy, childhood, and adolescence [6], represents one of the primary socialization spaces of children and adolescents and can thus decisively shape health behavior through role model behavior, rules, and support [7,8].

Although families occupy a crucial position in health promotion, access to them is difficult in practice. The family does not represent a spatially fixed and defined setting, such as the institutions of school, kindergarten, or a workplace [6]. Moreover, in most Western countries, families have a right to autonomy and privacy that must be respected when implementing health promotion interventions [6,9]. In these cases, it is not possible to oblige families to take health promotion actions. Here, work can only be done with families and not against them [6]. Existing institutional settings are suitable to reach and connect with families for this purpose. Pre-school childcare and schools are particularly suitable, as families are already integrated into these structures and children and adolescents are reached with their families in the early stages of their personal development [10].

The participation of families as addresses in the design and implementation of health promotion actions is helpful for increased acceptance and utilization [11]. The World Health Organization’s Global Action Plan for the Prevention of Noncommunicable Diseases states that prevention should begin in the first phase of life. Furthermore, they declare the empowerment and involvement of peoples and communities, among others, in the planning, implementation and evaluation of measures as another overarching principle [12].

In terms of participatory health promotion projects, the goals, planning, implementation, and evaluation of interventions should be developed in partnership [13]. Through this participatory approach, tailored services can be developed that are likely to be more efficient and effective [14]. There are various scientific models for understanding participation in health promotion [15]. Arnstein (1969), for example, posits the metaphor of a ladder in which participation only occurs when the decision has been fully transferred to people from the addressed living environment [16]. Other models, such as that of Simovska and Jensen (2009) [17], refer more to the diversity of actors involved and the process of participation [15]. This article is based on the stages of participation according to Wright et al. (2008), as the developmental nature of participation is made clear and lower stages of participation are considered. In the “non-participation” stage, decisions are made by people outside the addressee group without their participation. In the “preliminary stage of participation”, the interests of the addressees are heard, or they can participate in a non-binding decision-making process. In the “participation” stage, the addressees have a proportional decision-making competence or independent decision-making authority, but the project continues to be supervised by persons outside the addressee group. In the last stage, the “autonomous organization”, the responsibility for decision-making, planning and implementation lies exclusively with the addressees [11].

There are various approaches from the Anglo-American area within the broad spectrum of participatory health promotion and participatory research in health promotion projects, such as community-based participatory research (CBPR) [18] and participatory action research (PAR) [19]. Participatory methods and approaches are diverse, and an overview of applicable participatory approaches is needed, especially in family health promotion. There is a growing interest in family health promotion, but few experiences and systematic implementation examples of family participation in health promotion projects [3]. This study, therefore, addresses the questions: (a) which approaches, and actions are used in participatory family health promotion and prevention, and (b) what practical experience professionals describe regarding the application of these participatory approaches.

## 2. Materials and Methods

The search strategy, selection process and data analysis were based on the preliminary guidelines of the Cochrane Rapid Reviews Methods Group [20] and are presented below.

### 2.1. Search Strategy

In the period from September to October 2021, a systematic literature search was conducted in the databases PubMed, Web of Science and LIVIVO and supplemented by an additional hand search. The following search strategy was applied in PubMed and transferred to the other databases: ((((((((family*[Title]) OR parent*[Title]) OR pregnan*[Title]) OR Famili*[Title]) OR Elter*[Title]) OR schwanger*[Title])) AND (((((health promot*[Title]) OR prevent*[Title]) OR health*[Title])) OR (((Gesundheitsförder*[Title]) OR Prävent*[Title]) OR Gesundheit*[Title]))) AND (((((participat*[Title]) OR participatory research[Title]) OR participatory action research[Title]) OR participatory evaluation[Title]) OR participatory health research[Title]) OR community based participatory research[Title]) OR Partizipat*[Title]) OR partizipative Forschung[Title]) OR partizipative Qualitätsentwicklung[Title]) OR partizipative Gesundheitsforschung[Title]).

Publications were considered that were written in English or German, referred to industrialized nations and were published in the period 2010–2020. Included publications were on the topic of participatory health promotion or primary prevention, in which preliminary stages or stages of participation with families were applied along the lines of Wright et al. (2008). In addition, the family should be considered as a system and family health promotion should take place in primary socialization (pregnancy, birth, early childhood) or in secondary socialization (external care through day care, primary school, secondary school). Studies were excluded that had no participatory components, presented participation as mere participation in an offer, or took place outside the named socialization phases, such as in family care for the elderly or in the care of relatives. Apart from the existence of a full text, no specifications were made regarding the type of manuscript.

### 2.2. Selection Procedure and Data Analysis

**Figure 1:**
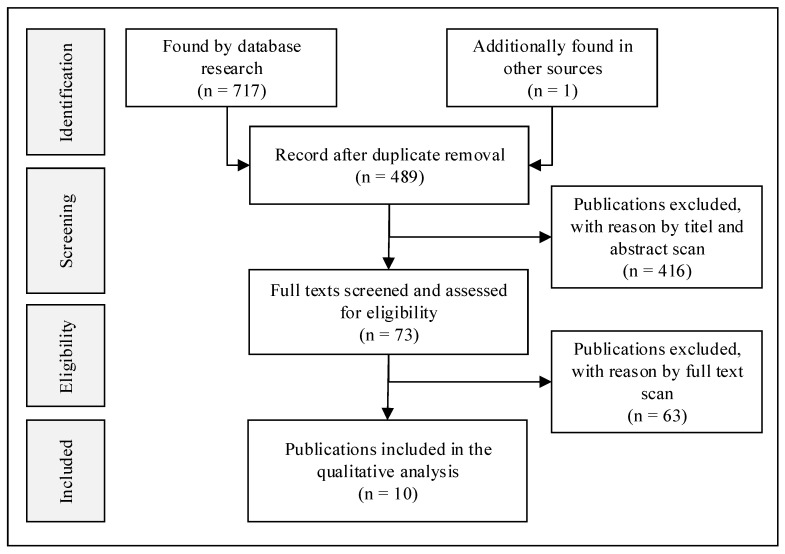
PRISMA flow diagram of included publications.

Study identification and data extraction were carried out independently by two research assistants. Figure 1 shows the identification process of the included studies. After an initial screening of title, abstract and full text, ten of the 718 publications found in the databases and by hand search were included in the data analysis. From these publications, the relevant data were extracted and tabulated. Relevant data included the differentiation of participatory approaches and reported experiences on effects and facilitating factors in the participatory process.

## 3. Results

The ten publications included different types of publications (four empirical studies, three research reports, three practice reports) and came from different countries (USA (7), DE (2), FI (1)). The health promotion interventions and the addressees of the projects were also different. Table 1 gives an overview of the publications included.

The participatory approaches and methods of the projects and their relation to the research questions are discussed below. Subsequently, the practical experiences in the use and application of the contributions are presented under the identified topics of the observed effects and the described success factors for family participation. For a uniform clustering, the described inhibiting factors were reformulated into facilitating factors.

### 3.1. Participatory Approaches and Methods Used

To enable an overview and comparability, the participatory approaches and methods described in the included publications were differentiated according to six criteria concerning the research question, and are presented below. Table 2 shows an overview of the information collected.

#### 3.1.1. Participatory Approach

The seven US publications used the CBPR approach, which aims at creating structures for the participation of the addressees in the research process and shared decision-making power among the actors involved [31]. Ferré et al. (2010) also reported a move towards a community-led participatory research (CPPR) approach. The three European publications referred to participatory action research (PAR) [28], or a participatory approach in general, without defining the term precisely [27,29].

#### 3.1.2. Theory-Based

A total of seven publications referred to a theory or model. They either referred to participation, such as the citizen health care model [21,23,25,27], or to the form of intervention used [24,30], such as the community health worker model in the publication by Garcia et al. (2012) and Carney et al. (2012).

#### 3.1.3. Involved Actors

Parents were explicitly mentioned as involved actors in five publications, families in three publications and children in one publication. These publications referred to school, kindergarten, or community settings. Berge et al. (2016) and Carney et al. (2012) not only explicitly named families and parents, but also referred to the community in which they live. Some mentioned the community without further differentiation of the actors involved [23,26,30].

#### 3.1.4. Form of Participation

Three of the ten publications included only descriptions of preliminary stages of participation, such as informing, listening, or involving the addressees [22,28,29]. Johnson-Shelton et al. (2015) and Schäfer and Bär (2019) exclusively indicated forms of actual participation. Five publications described preliminary stages and actual participation [21,23,24,26,30].

#### 3.1.5. Project Phase

Following the public health action cycle [32], all publications describe a participation of the addressees in the project development phase. Eight publications describe a participation in the implementation phase [21,22,23,24,25,26,27,28] and seven in the analysis [21,23,24,26,27,29,30] and evaluation phase [21,22,23,24,25,26,27]. In total, five publications refer to participation in all project phases [21,23,24,26,27].

#### 3.1.6. Participatory Methods

Participatory methods used can be divided into four categories. Seven times each, in different combinations, it was described that regular meetings (with the community, within the research team) were held [21,22,23,24,25,26,27], interviews and focus group discussions took place [21,22,24,26,27,29,30], and advisory board, action group or research group with the addressees were formed [21,23,24,25,26,27,30]. Six publications described that events, such as kick-off events, conferences or workshops, were held [21,23,26,27,28,30].

### 3.2. Practical Experience in the Use and Application of Participatory Methods

Table 3 provides an overview of the described experiences with the participatory approaches and measures of family health promotion in the publications considered. Reported effects and described facilitating factors were defined as criteria for the described experiences.

#### 3.2.1. Reported Effects/Impact of the Participatory Approach

Various observations and experiences regarding the impact of the participatory approach were reported. Most publications mention the formation and strengthening of partnerships [21,22,23,24,25,26,28,30]. It was often emphasized that the participatory approach chosen should include addressee-specific perspectives and aspects [24,25,26,27,30]. Four publications described that participation had an influence on the acceptance/satisfaction with the intervention [21,28,29,30], that innovative actions were developed [25,26,29,30], and that participants acquired competences and/or knowledge [24,26,27,28]. Four of the ten publications described that participants were satisfied with the process [28] or felt equally involved [30], with three publications mentioning both [21,24,27]. Other aspects were the motivation for (further) participation [24,26,30], an observed positive influence on the desired behavior change and an improvement in health [22,30].

#### 3.2.2. Success Factors for Family Participation

Most success factors are found in process design. The most frequently described factor is respectful interaction and communication on an equal level [21,22,23,24,26,27,30]. Half of the publications describe the relevance of a common goal or the exchange of respective goals [24,25,26,27,30]. The identification and use of existing resources is also highlighted as helpful [21,23,24,25,26]. Promotional for family participation is the open participation and involvement opportunity, especially the flexibility in the scope of participation [21,23,28]. Sormunen et al. (2013) emphasized compatibility with the family; for example, childcare should be offered, or activities planned for the whole family. Three other publications describe that it is beneficial to involve the families’ environment [24,25,26] and two how helpful it is to address the addressees broadly and at a low threshold [29,30]; for example, by advertising via social media, emails, and phone calls and by addressing the addressees in their native language.

Some publications refer to general framework conditions such as financial and human resources [23,24,27,29], which are particularly relevant for enabling a successful participation design. More time needed for participatory processes is emphasized several times [23,24,27]. More than half of the publications refer to process facilitation, describe the importance of structure and coordination [23,25,26,30] and flexibility in the process [23,24,25,27]. Two publications also describe staff training as beneficial [29,30].

Regarding specific aspects of participation, the commitment of the participants and their desire for change are mentioned several times as beneficial [23,24,25,27,28]. Garcia et al. (2012) and Johnson-Shelton et al. (2015) emphasized the importance of balancing research and action in the process (to meet families’ desire for change). The fact that empowering the addressees had a positive impact on the process is described three times [25,27,28]. Sormunen et al. (2013) emphasized that successful participation requires that the addressees have the opportunity to influence the process and that an existing culture of participation in the environment (in the institution or community) can have a positive impact on participation. They further express that open access, sharing and use of data is beneficial for partnership and participation [23,24,25].

## 4. Discussion

### 4.1. Discussion of Methods

The geographical imbalance of the search hits could indicate possible limitations in the search terms used, e.g., that the keywords used only relate to research practice in the USA. Clar and Wright (2020) emphasize that participatory approaches are not considered research by many practitioners who use these approaches. As a result, some experiences with using participatory approaches may not be found in academic journals and standard databases. Given the current data situation, future methodological approaches may benefit from following the recommendation for limited use of grey literature and supplementary internet research in the preliminary guide to the rapid review method [20].

The publications analyzed describe their participatory components in varying depth and degrees, which, according to [33] or [34], is a characteristic of the entire field of participatory research in health promotion. Gathering information is difficult because aspects of participation are not described in detail. As a result, individual project experiences are lost, and potentially relevant contributions may be excluded due to a lack of information. How participation was organized in the projects included here could not be adequately clarified. However, the tabular presentation enabled a helpful systematization and comparability and proved useful for a first overview. With a clearer understanding of participation in the general research culture, further rapid reviews may provide more in-depth insights in the future. In addition, telephone contact with authors could provide a more concrete representation of the reality of future projects [34].

### 4.2. Discussion of the Results

#### 4.2.1. Prevalence of Family Participatory Approaches

Overall, the number of included publications exemplifies that the participation of families in the process of health promotion projects is not yet widespread and has not been sufficiently investigated scientifically. In the large number of excluded publications, participation was often equated with pure participation and not with a decision-making competence of the addressees in the design of a health-promoting offer. For scientific research on participation in family health promotion, the development of a uniform understanding of participation is necessary, as is repeatedly demanded in the participatory research culture [34]. A common definition and differentiation would make the research comparable and enable more targeted information to be collected on the use of participatory family health promotion.

The accumulation of hits in the Anglo-American area is partly due to a longer history of development of participatory research in North America [35]. In the USA, the approach of CBPR is widespread, while European publications use different and partly undefined participatory approaches. Two further research aspects would be worthwhile for the European area. First, the identification of factors that lead to the low application of elsewhere common participatory approaches such as CBPR, currently is hardly used in European participatory health promotion. Second, an in-depth academic exchange on the benefits and effects of participatory approaches in family health promotion should be started/encouraged. Both would be conceivable within the framework of Delphi studies. This could support the already existing efforts in the development of uniform participatory approaches in Europe. For example, a group of the German-speaking network PartNet is in the process of translating and testing the CBPR model [36]. This development is likely to be influenced by the trend towards “participation” in research and health promotion, although it is still undetermined whether the research culture in general will change or whether participatory research will continue to prevail as a specific approach [37].

#### 4.2.2. Family Participation to Prevent Chronic Diseases

Some publications describe actions to reduce risk factors for chronic diseases. Similar to the WHO Global Action Plan for the Prevention and Control of NCDs 2013–2020, different determinants were addressed. Three publications describe projects dealing with nutrition and obesity [21,25,30], in the intervention of one publication the goal is to reduce passive smoking as a risk factor for the development of chronic diseases in childhood [29]. CBPR is emphasized in two publications as an approach that allows for the development of culturally sensitive interventions for disease prevention (also of chronic diseases) [23,30]. Participation enables the development of interventions individually for each community, i.e., through understanding the community’s risk factors appropriate solutions can be found together.

#### 4.2.3. Possible Applications for Participation in Family Health Promotion

Families consist of different family members who are to be reached in participatory health promotion [3]. The results in the included publication emphasize that the attitude towards the participation of children and adolescents needs development, as in most of the publications they are not considered in the participatory process. This passive role does not give them the opportunity to help shape the offers intended to influence their own health attitudes and behaviors. Michaelson et al. (2021) show that many conceptual and theoretical models of the family in health promotion do not recognize children as active agents. Concerns regarding how children and young people may negatively affect the quality of research and an underestimation of children’s competencies contribute to the exclusion of children and young people in health promotion models [14,38,39].

The results show that for large groups the levels “informing” and “listening” were used, and for defined small groups members were given decision-making competencies and powers. This illustrates that different levels of participation can be present during a project and that these are oriented towards the circumstances and existing possibilities of the project. Preliminary stages of participation and actual participation thus have their place in the project process. However, when working with the addressees, regular checks should be made on how decision-making powers and responsibilities can be transferred to them and whether the opportunities for participation are being fully maximized [13]. A variety of methods are reported in the publications, ranging from regular meetings to events. Some of the descriptions indicate how participation was ensured, but not how the methods were implemented. There is a need for research and presentation regarding the application of the individual methods and clear criteria for participation.

A suitable project phase for the participation of family actors seems to be the development phase. In all publications, participation took place in this phase. Participation here and in the preceding joint analysis phase is necessary for the course of the project, as this can lead to tailored actions that positively influence effectiveness and sustainability [14]. It can be assumed that successful participation in the early project phases lays the foundation for participation in the subsequent phases. It is critical to note that family actors are less often involved in the final evaluation phase of the project. It is often unclear how, and to what extent, they will be informed about the results produced or involved in further steps. The question here is whether this is only of interest to the researchers, as the additional perspectives mean that the best possible research methods and the long-term benefits of the research are recognized by the addressees [31], or whether the family actors are interested, or gain added value by participating in the evaluation. Incentives may motivate addressees’ participation, and is practiced in countries with a long tradition of participatory health research.

#### 4.2.4. Effects of the Participatory Approach

The results show that there are many positive effects on different levels through family participation. The fact that family participation strengthens and forms new partnerships coincides with the general impact of participatory projects on relationships [40]. It expands resources and paves the way for later collaborations. The emphasis on this aspect, in almost all publications analyzed, illustrates the attractiveness of this aspect for the institutions involved. The effects described are closely related to the type and extent of participation. For example, groups of authors report a feeling of equal participation if the addressees were closely involved in the process as part of a formed action or research group [21,24,27,30]. Others emphasize the acceptance of actions after information and or involvement of larger groups (community or similar) [21,28,29].

The preliminary stage of participation can already have positive effects, and since the research and health promotion culture in Europe is still not very participatory, applying this stage would form a foundation for participation and health promotion. Higher acceptance of actions and a decrease of health inequalities, precise recommendations for action are needed. It is unclear how the complexity in the development of effects [41] can be assessed.

#### 4.2.5. Similar Success Factors as for Participatory Processes in General

A successful process requires the inclusion of families in health promotion projects, fundamental aspects such as respectful interaction, communication as equals, and agreement on a goal. These factors, and the need for additional resources (financial, personnel, time), do not differ from health promotion projects [42] but gain even more relevance under the aspect of participation. The result that communication and interaction as equals are mentioned particularly frequently illustrates that power imbalances and inequalities in the setting should be critically reflected upon [43]. These represent a central limitation to participation in projects [44].

The engagement of the participants through their desire for change illustrates the relevance and opportunity that are commonly defined goals. A balance of research and action needs to be considered in the planning and implementation of projects. This desire for action presumably correlates to the challenge of joint research and the evaluation of actions and processes described above.

#### 4.2.6. The Importance of Empowerment and Flexibility

Empowerment and participatory attitudes (participatory culture in the setting) are a core element of participatory health research, yet are only addressed in a few publications [40]. It is possible that this lack of reporting results from an incorrect perception that families are sufficiently empowered in their settings, or a lack of awareness for the need for empowerment. This is an important aspect to consider when planning and implementing participatory projects with families. Flexibility in the nature and extent of participation is even more important for families than for other target groups. As Sormunen et al. (2013) have described, participation opportunities must be compatible with the everyday life of the individual family.

## 5. Conclusions

In conclusion, the following aspects can be summarized with respect to the research questions:

1. There are only a few publications on participatory family health promotion projects, and there is a great need for theoretical and methodological development, especially outside the Anglo-American area, i.e., Europe.
2. Form and method of participation must be adapted to individual circumstances, and continuous consideration should be given to how the highest possible form of participation can be achieved.
3. Participation in family health promotion leads to effects on different levels, including strengthening partnerships and a higher acceptance of actions, and can be used to develop interventions that reduce chronic diseases.
4. As with other participation processes, particularly suitable framework conditions and attitudes contribute to success, whereby flexibility in the form of participation is of particular importance in family participation.

The participatory approach and its impact are complex, as is the behavioral change that results from participatory action. This is probably why this outcome is so rarely explored in publications. This review shows that the participatory approach nevertheless has many effects that will, over time, trigger behavior changes in the family and the respective environment or community. The approach is, therefore, very promising because behavior can be influenced in the entire living environment.

## Figures and Tables

**Table 1:** Overview of included publications.

Authors [Citation]	Year	Country	Manuscript Type	Health Promoting Actions and Addressees
Berge et al. [21]	2016	US	S	Family-oriented actions to prevent childhood obesity as a risk factor for chronic diseases
Carney et al. [22]	2012	US	S	Project on nutrition in Hispanic families
Ferré et al. [23]	2010	US	P	Multi-phase project on the topic of pregnancy health
Garcia et al. [24]	2012	US	P	Community school-based intervention for Latino youth, including depression prevention
Johnson-Shelton et al. [25]	2015	US	P	Various school and community health promotion programs for the prevention of childhood obesity
Jones et al. [26]	2010	US	P	Several strategies for risk communication about prematurity
Schäfer & Bär [27]	2019	DE	R	Improving equal opportunities for families with children of day-care age through peer researchers (parents)
Sormumen et al. [28]	2013	FI	S	School health interventions to strengthen health education
Weinmann et al. [29]	2018	DE	R	Awareness campaign on the consequences of passive smoking for children
Wieland et al. [30]	2016	US	S	Development of health promotion actions for immigrant families in relation to nutrition and obesity as a risk factor for chronic diseases

Legend: S = empirical study, R = research report, P = practice report.

**Table 2:** Approaches and methods of participatory family health promotion and prevention in the publications considered.

Publication (Author & Year) [Citation]	Approach	Theory-Based	Participating Actors of the Addressee Group	Form of Participation	Project Phase				Participatory Method
					I	II	III	IV	
**Berge et al. (2016)** [21]	CBPR	+ Citizen Health Care Model	+ Citizen Action Group (CAG) (interested parents of the community, university researchers) + Community members	+ Preliminary stage: Hearing and informing members/families from the community. + Participation: CAG has decision-making authority	X	X	X	X	+ Initial launch event (Families from the district) + Citizen Action Group (CAG) + Interviews (conducted by CAG participants with community members) + Regular weekly CAG meetings
**Carney et al. (2012)** [22]	CBPR	+ Community Health Worker	+ Community group + Families	+ Preliminary stage: inform and involve families and community members	-	X	X	X	+ Interviews (with the participation of community members) + Regular monthly community meetings (families, community members)
**Ferré et al. (2010)** [23]	CBPR and Community-partnered Participatory Research (CPPR)	+ Community assets model	+ Community advisory board (CAB) + Community	+ Preliminary stage: inform and involvement of community + Participation: Decision-making authority Research team (phase 1), CAB (phase 2), community organization (phase 3)	X	X	X	X	Phase 1 i.e.,: + Community advisory board (CAB) + Regular meetings (community meetings, CAB and other partners in community locations)Phase 2 i.e.,: +Event: community conference (Community members)Phase 3 i.e.,: + Events: Conferences, workshops, symposia etcetera (community members)
**Garcia et al. (2012)** [24]	CBPR	+ Community Health Worker Model + Ecological model + Complexity theory	+ Research team (professionals from school and community-based clinic, families, university) + Participating families	+ Preliminary stage: Informing andListening to families and research team + Participation: Decision-making competence of research team	X	X	X	X	+ Research team + Regular weekly/monthly meetings (research team) + Focus group discussions (with parents)
**Johnson-Shelton et al. (2015)** [25]	Multilevel-Partnership nach CBPR	+ Multilevel CBPR model (hybrid model) + Organizational learning	+ Communities and Schools Together Project (CAST) partnerships (school district and staff, parents and families of primary schools, community of NGO groups, scientific community)	+ Participation: CAST partnerships & working groups	-	X	X	X	+ Parent Advisory Council (elementary school parents) + Regular meetings (CAST partnerships) + Events: working groups (with CAST partners)
**Jones et al. (2010)** [26]	CBPR	-	+ Community members (including pregnant women) as equal stakeholders	+ Preliminary stage: listening to community members + Participation: decision-making authority lies with project team, steering and sub-committees	X	X	X	X	+ Project team (local community and non-community members) + Steering committee (individuals from various localcommunity sectors) + Subcommittee (members of steering committee) + Events: Training
**Schäfer & Bär (2019)** [27]	Participatory data evaluation (no defined term)	+ Action model of action research + Emancipatory action research	+ Parents of children of kindergarten age	+ Participation: Decision-making competence lies with research team	(X)	(X)	(X)	X	+ Research workshops (including interviews of additional parents) + research team (parents and researchers) + Workshop and regular meetings in research workshops
**Sormunen et al. (2013)** [28]	PAR, setting approach	-	+ Pupils + Parents + Teachers + School management	+ Preliminary stage: Involvement of pupils, parents, teachers, school management, informing parents	-	X	X	-	+ Events: Parent conference, health-related workshops/topic evenings (parents, pupils)
**Weinmann et al. (2018)** [29]	Participatory approach (no defined term)	-	+ Parents of the addressee group	+ Preliminary stage: Involvement of parents	X	X	-	-	+ Interviews (with caregiver of children) + Focus groups (with caregiver of children)	
**Wieland et al. (2016)** [30]	CBPR	+ Social cognitive learning theory	+ Community members from each participating immigrant group of immigrant families	+ Preliminary stage: Listening to other community members + Participation: Decision-making competence Research team	X	X	-	-	+ Study team, working group (community members, health scientists) + Focus groups (members of local migrant communities) + Events: Training on how to implement the intervention in their own community

Legend: I = Analysis; II = Development; III = Implementation; IV = Evaluation. X—Form of participation is described for this project phase. (X)—Form of participation is indicated in the publication and/or described in connection with the respective project phases. -—Form of participation is not described for this project phase. + Aspect is mentioned in the article.

**Table 3:** Described experiences [reported effects & described facilitating factors] with the participatory approaches and measures of family health promotion in the publications considered.

Publication *(Author & Year)* *[Citation]*	Reported Effects or Observed Developments										Described Experiences with Facilitating Factors																	
	*Formation/Strengthening of New partnerships*	*Inclusion of Addressee-Specific Perspectives/Aspects*	*Competence and/or Knowledge Enhancement*	*Development of Innovative Measures/Products*	*Satisfaction with/Acceptance of the Intervention*	*Participating Stakeholders Satisfied with the Process*	*Sense of (Equal) Participation*	*Motivation for (Further) Participation*	*Desired Behavior Change*	*Improving Mental/Physical Health*	*Process Design*							*Framework and Process Support*					*Specific Participation Aspects*					
											*Interaction/Communication*	*Joint Goal*	*Resource Use*	*Open Participation Opportunities*	*Family Compatibility*	*Involvement of Environment*	*Broad/low-Threshold Approach*	*Financial & Human Resources*	*Time*	*Structure & Coordination*	*Flexibility*	*Trained Personnel*	*Commitment & Desire for Change*	*Access to Data and Dissemination*	*Empowerment*	*Balance Research/Action*	*Opportunities for Influence*	*Participatory Atmosphere*
Berge et al. (2016) [21]	✓	-	-	-	✓	✓	✓	-	-	-	✓	-	✓	✓	-	-	✓	-	-	-	-	-	-	-	-	-	-	-
Carney et al. (2012) [22]	✓	-	-	-	-	-	-	-	✓	✓	✓	-	-	-	-	-	-	-	-	-	-	-	-	-	-	-	-	-
Ferré et al. (2010) [23]	✓	-	-	-	-	-	-	-	-	-	✓	-	✓	✓	-	-	-	✓	✓	✓	✓	-	✓	✓	-	-	-	-
Garcia et al. (2012) [24]	✓	◦	✓	-	-	✓	✓	✓	-	-	✓	✓	✓	-	-	✓	-	✓	✓	-	✓	-	✓	✓	-	✓	-	-
Johnson-Shelton et al. (2015) [25]	✓	✓	-	✓	-	-	-	-	-	-	-	✓	✓	-	-	◦	-	-	-	✓	✓	-	✓	◦	-	✓	-	-
Jones et al. (2010) [26]	✓	✓	✓	✓	-	-	-	✓	-	-	✓	✓	✓	-	-	✓	-	-	-	✓	-	-	-	-	✓	-	-	-
Schäfer & Bär * (2019) [27]	-	✓	✓	-	-	◦	◦	-	-	-	✓	✓	-	-	-	-	-	✓	✓	-	✓	-	✓	-	✓	◦	-	-
Sormunen et al. (2013) [28]	✓	-	✓	-	✓	✓	-	-	-	-	-	-	-	✓	✓	-	-	-	-	-	-	-	✓	-	✓	-	✓	✓
Weinmann et al. (2018) [29]	-	✓	-	✓	✓	-	-	-	**	**	-	-	-	-	-	-	✓	✓	-	-	-	✓	-	-	-	-	-	-
Wieland et al. (2016) [30]	✓	✓	-	◦	◦	-	◦	✓	◦	◦	✓	✓	-	-	-	-	-	-	-	✓	-	✓	-	-		-	-	-
Sum	8	6	4	4	4	4	4	3	2	2	7	5	5	3	1	3	2	4	3	4	4	2	5	3	3	2	1	1
											10							8					6					

✓ Aspect is presented in connection with the described measures or highlighted as beneficial. ◦ Aspect is stated and/or described in connection with the overall project/previous measures or as part of the process. - Aspect is not described. * The facilitating factors refer to the described aspect of the process “joint data evaluation”. ** Aspect to be further examined.

## Data Availability

Not applicable.
